# Serum Amyloid A Is a Biomarker of Disease Activity and Health-Related Quality-of-Life in Patients with Antineutrophil Cytoplasmic Antibody-Associated Vasculitis

**DOI:** 10.1155/2020/8847306

**Published:** 2020-12-16

**Authors:** Taejun Yoon, Sung Soo Ahn, Juyoung Yoo, Jason Jungsik Song, Yong-Beom Park, Sang-Won Lee

**Affiliations:** ^1^Department of Medical Science, BK21 Plus Project, Yonsei University College of Medicine, Seoul, Republic of Korea; ^2^Division of Rheumatology, Department of Internal Medicine, Yonsei University College of Medicine, Seoul, Republic of Korea; ^3^Institute for Immunology and Immunological Diseases, Yonsei University College of Medicine, Seoul, Republic of Korea

## Abstract

Serum amyloid A (SAA) is one of the acute phase proteins synthesized in hepatocytes and secreted by various inflammation or infectious stimuli. We investigated the clinical implication of measuring SAA in patients with antineutrophil cytoplasmic antibody- (ANCA-) associated vasculitis (AAV). Seventy-five patients who had been classified as AAV and enrolled in our prospective observational cohort for AAV patients were included. Clinical and laboratory data were obtained on the day of blood sampling, and SAA was measured by ELISA kits. Birmingham Vasculitis Activity Score (BVAS) and Short-Form 36-Item Health Survey (SF-36) were assessed for disease activity and health-related quality-of-life (HRQoL) measures. We stratified patients into having high BVAS when the BVAS was over the median values, and those with either low SF-36 PCS or low SF-36 MCS were defined as having poor HRQoL. Multivariate logistic regression analysis was conducted to estimate independent predictors of high BVAS. The relative risk (RR) was analyzed using the contingency tables and the chi-squared test. SAA was positively correlated with BVAS (*r* = 0.642) and FFS (*r* = 0.367) and was inversely correlated with both the SF-36 physical component summary (*r* = −0.456) and mental component summary scores (*r* = −0.394). Furthermore, SAA was significantly correlated with acute phase reactants ESR (*r* = 0.611) and CRP (*r* = 0.629). Patients with high BVAS exhibited significantly higher SAA than those with low BVAS (1317.1 ng/mL vs. 423.1 ng/mL). In multivariable logistic regression analysis, serum albumin (odds ratio (OR) 0.132) and SAA > 1173.6 ng/mL (OR 15.132) were independently associated with high BVAS. The risk of having high BVAS and poor HRQoL in patients with SAA > 1173.6 ng/mL was higher than in those with SAA ≤ 1173.6 ng/mL (RR 3.419 and 1.493). Our results suggest that SAA might be a useful biomarker in assessing disease activity and HRQoL in AAV.

## 1. Introduction

Antineutrophil cytoplasmic antibody- (ANCA-) associated vasculitis (AAV) is a group of the systemic vasculitides affecting small vessels such as microscopic polyangiitis (MPA), granulomatosis with polyangiitis (GPA), and eosinophilic granulomatosis with polyangiitis (EGPA), which exhibit necrotizing vasculitis in arterioles, capillaries, and venules [1]. However, heterogeneous clinical phenotypes are present between the three diseases: MPA primarily provokes rapidly progressive crescentic glomerulonephritis and diffuse alveolar hemorrhage, whereas GPA mainly forms granulomas and affects the upper and lower respiratory tracts [1, 2]. By contrast, EGPA is composed of 3 phases such as allergic, eosinophilic, and vasculitis phases and is characterized by asthma, sinusitis, peripheral eosinophilia, eosinophil infiltration, and organ damages in lungs and nerves [3].

Birmingham Vasculitis Activity Score (BVAS) has been improved over the decades, and its 3rd version is now most commonly used for assessing the disease activity of AAV [4]. Since BVAS covers a wide range of nine systemic categories with differently weighted scores based on the severity of each symptom, it has been considered the most reliable tool to assess AAV activity to date [5]. However, since BVAS includes not only the cross-sectional clinical features but also the chronic clinical features such as lung fibrosis and renal dysfunction, there is a difficulty using BVAS as an indicator of cross-sectional activity or severity of AAV [5]. So far, we have demonstrated the clinical significance of several AAV activity-related indices consisting of serum concentrations of endogenous proteins or equations of laboratory data for predicting the cross-sectional activity of AAV based on BVAS [6, 7]. Although the clinical utility of these indices may not exceed that of BVAS, owing to the paucity of reliable biomarkers available, it is meaningful to search for novel markers that hold clinical implications in AAV which are expected to possess a complementary role in assessing AAV activity.

Serum amyloid A (SAA) is one of the typical acute phase proteins synthesized in hepatocytes and secreted by various inflammation or infectious stimuli [8, 9]. The production and secretion of SAA from the liver are accelerated by proinflammatory cytokines such as interleukin- (IL-) 1, IL-6, tumor necrosis factor (TNF), interferon-*γ*, and transforming growth factor-*β* (TGF-*β*) [10]. Therefore, it could be theoretically assumed that SAA may be closely correlated with the amount of the inflammatory burden of autoimmune disorders. In this context, previous studies elucidated that SAA was significantly correlated with the activity of systemic vasculitides such as Takayasu arteritis, Behcet's disease, and Henoch-Schönlein purpura [11–13]. Furthermore, SAA has also been reported to be a potential biomarker of different types of lung disorders and inflammatory environment and is useful in assessing disease activity of systemic lupus erythematosus [14–16]. However, to the best of our knowledge, there was no study that reported the association between SAA and clinical and laboratory features of AAV. Hence, in this study, we investigated the utility of measuring SAA from a prospective cohort of AAV patients.

## 2. Materials and Methods

### 2.1. Study Population

In this study, we included 75 AAV patients who had been enrolled in the Severance Hospital ANCA-associated VasculitidEs (SHAVE) cohort from November 2016 to May 2019. The SHAVE cohort is a prospective observational cohort of patients with MPA, GPA, and EGPA, which began in November 2016 in a tertiary referral center in South Korea. All patients were classified as AAV at the Division of Rheumatology, Department of Internal Medicine, Severance Hospital. They all fulfilled the 1990 American College of Rheumatology classification criteria for EGPA, the 2007 European Medicines Agency algorithm for AAV and polyarteritis nodosa (the 2007 EMA algorithm), and the 2012 revised International Chapel Hill Consensus Conference Nomenclature of Vasculitides [1–3]. Based on the entry requirement of the 2007 EMA algorithm, AAV patients, who were accompanied by chronic infection including hepatitis B or C virus infection, malignancies, or secondary vasculitis features related to autoimmune diseases, were excluded [2]. The ethical permission regarding the study was approved by the Institutional Review Board of Severance Hospital (4-2016-0901).

### 2.2. Clinical and Laboratory Data

Demographic data included age, gender, and disease duration. The clinical manifestations were counted based on the items of BVAS version 3, and laboratory tests including ANCAs were performed. Four AAV-specific indices were assessed: the BVAS version 3 for disease activity [5]; the five-factor score (2009) for prognostic evaluation [17]; the Korean version of the Short-Form 36-Item Health Survey (SF-36) for health-related quality-of-life (HRQoL) [18]; and the vasculitis damage index (VDI) for organ injury or damage [19]. The BVAS was calculated for all of the patients by evenly applying BVAS version 3 to unify the scoring system. On the same day of clinical and laboratory data obtainment, whole blood was drawn from each patient upon consent and was immediately centrifuged to isolate sera which were then stored at –80°C. SAA was measured from stored sera with ELISA kits from Invitrogen (Waltham, MA, USA) according to the manufacturer's instruction.

### 2.3. Definition of High BVAS and Poor HRQoL

We stratified patients into having high BVAS when the BVAS was over the median values and low SF-36 physical component summary (SF-36 PCS) and SF-36 mental component summary (SF-36 MCS) when the values were lower than the median values. Patients were defined as having poor HRQoL when they had either low SF-36 PCS or low SF-36 MCS.

### 2.4. Statistical Analyses

All statistical analyses were conducted using MedCalc statistical software version 19.2 (MedCalc Software, Ostend, Belgium). Continuous variables were expressed as mean with standard deviation, and categorical variables were expressed as number (percentage). The correlation coefficient between the two variables was obtained using the Spearman correlation analysis. Significant differences in categorical variables between the two groups were analyzed using the chi-squared test and Fisher's exact test. Significant differences in continuous variables between the two groups were compared using Student's *t*-test. The receiver operating characteristic (ROC) curve was used to identify the optimal cut-off value and the area under the ROC curve (AUROC) of SAA in differentiating between the high and low BVAS, as well as poor and high HRQoL. The multivariable logistic regression analysis which included variables with *p* values less than 0.05 on the univariable analysis was conducted to assess the odds ratio (OR) of variables in predicting high BVAS. The relative risk (RR) was analyzed using the contingency tables and the chi-squared test. *p* values less than 0.05 were considered statistically significant.

## 3. Results

### 3.1. Baseline Data and Association between SAA with AAV-Specific Variables and Laboratory Data

Among the 75 patients included, MPA (50.7%) was the most common diagnosis, followed by GPA and EGPA. The mean age and disease duration of the patients were 58.9 and 18.2 months, and 26 (34.7%) of the patients were male. The mean value of BVAS, FFS, VDI, SF-36 PCS score, and SF-36 MCS score was 9.6, 1.3, 3.2, 49.9, and 56.8, respectively. Pulmonary manifestation (64.0%) was the most common clinical feature present, and ANCA was detected in 49 (65.3%) of patients (Table 1).

In correlation analysis, SAA was revealed to be positively correlated with BVAS (*r* = 0.642) and FFS (*r* = 0.367) and was negatively correlated with both the SF-36 PCS (*r* = −0.456) and MCS scores (*r* = −0.394). In addition, SAA was significantly correlated with white blood cell count, hemoglobin, platelet count, blood urea nitrogen, creatinine, total protein, and serum albumin, along with acute phase reactants of erythrocyte sedimentation rate (ESR) (*r* = 0.611) and C-reactive protein (CRP) (*r* = 0.629). Moreover, although BVAS was significantly correlated with VDI, SAA was not meaningfully correlated with VDI (Table 2).

### 3.2. Comparison of Clinical and Laboratory Features in Patients with High and Low BVAS

We divided our patients into two groups of the high BVAS group (*N* = 38) and the low BVAS group (*N* = 37). There were no differences in AAV variants between the two groups. However, patients with high BVAS had shorter disease duration and lower SF-36 PCS and MCS scores than those with low BVAS. Among clinical manifestations, patients with high BVAS presented general, pulmonary, and renal manifestations more frequently than the low BVAS group. Myeloperoxidase- (MPO-) ANCA and ANCA positivity were detected more often in patients with high BVAS. In regard to laboratory data, patients with high BVAS showed higher white blood cell count, platelet count, blood urea nitrogen, creatinine, ESR, and CRP but lower hemoglobin, total protein, and serum albumin compared to patients with low BVAS. Patients with high BVAS had significantly higher SAA than the low BVAS group (1317.1 ng/mL vs. 423.1 ng/mL, *p* < 0.001) (Table 3).

### 3.3. Comparison of SAA Levels Based on the Presence of Organ Involvement

We also investigated differences in SAA depending on the presence or absence of each organ involvement. Patients with general, mucous membrane and eye, pulmonary, and renal manifestations exhibited higher SAA levels than those without (Table 4). There was no difference in SAA levels regarding cutaneous; ear, nose, and throat; cardiovascular; and nervous system involvement.

### 3.4. Independent Predictors of High BVAS in Logistic Regression Analysis

**Figure 1:**
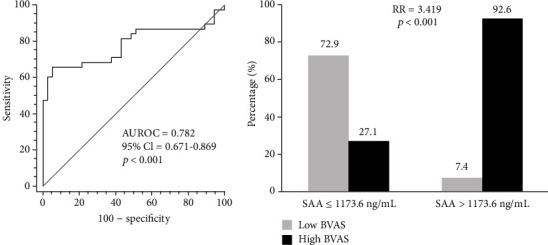
Optimal cut-off of SAA in predicting high BVAS and the relative risk according to the cut-off value. The optimal cut-off of SAA for predicting high BVAS was 1173.6 ng/mL, and patients with SAA > 1173.6 ng/mL exhibited a significantly higher risk of having high BVAS than those with SAA ≤ 1173.6 ng/mL. SAA: serum amyloid A; BVAS: Birmingham Vasculitis Activity Score; AUROC: area under the receiver operating characteristic curve; CI: confidence interval; RR: relative risk.

The optimal cut-off of SAA for predicting high BVAS was obtained as 1173.6 ng/mL using the ROC curve (AUROC 0.782, 95% confidence interval (CI) 0.671-0.869, and *p* < 0.001) with a sensitivity of 65.8 and specificity of 94.6 (Figure 1). When we divided AAV patients into the two groups based on this cut-off of SAA, patients with SAA > 1173.6 ng/mL exhibited a significantly higher risk of having high BVAS than those with SAA ≤ 1173.6 ng/mL (RR 3.419, *p* < 0.001) (Figure 1).

In the univariable logistic regression analysis, high BVAS was associated with all laboratory variables included except P-ANCA. However, in the multivariable logistic regression analysis, high BVAS was only associated with SAA > 1173.6 ng/mL (OR 15.132, 95% CI 2.656-86.196, and *p* = 0.002) and serum albumin (OR 0.132, 95% CI 0.032-0.551, and *p* = 0.006) (Table 5).

### 3.5. Comparison of HRQoL Measures according to SAA Levels

**Figure 2:**
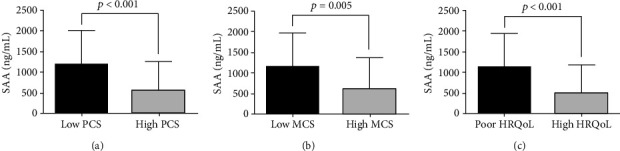
Comparison of SAA levels according to HRQoL measures. Patients with low SF-36 PCS (a), low SF-36 MCS (b), and poor HRQoL (c) had significantly higher SAA compared to those without. SAA: serum amyloid A; HRQoL: health-related quality-of-life; SF-36: Short-Form 36-Item Health Survey; PCS: physical component summary; MCS: mental component summary.

**Figure 3:**
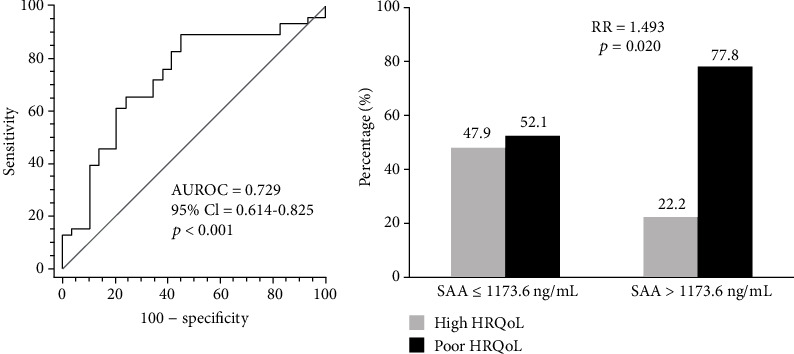
AUROC of SAA in predicting poor HRQoL and relative risk. The AUROC of SAA in predicting poor HRQoL was 0.729, and the risk of having poor HRQoL was significantly higher in patients with SAA > 1173.6 ng/mL than in those with SAA ≤ 1173.6 ng/mL. AUROC: area under the receiver operating characteristic curve; SAA: serum amyloid A; HRQoL: health-related quality-of-life; CI: confidence interval; RR: relative risk.

Next, we compared SAA levels in the patients according to measures of HRQoL. SAA was found to be significantly higher in patients with low PCS, low MCS, and poor HRQoL (Figure 2). Similarly, the optimal cut-off of SAA in differentiating poor and high HRQoL was >111.1 ng/mL, with an AUROC of 0.729 (95% CI 0.614-0.825, *p* < 0.001) and a sensitivity of 89.1 and specificity of 55.2 (Figure 3). The risk of having poor HRQoL was also significantly higher in patients with SAA > 1173.6 ng/mL than in those with SAA ≤ 1173.6 ng/mL (RR 1.493, *p* = 0.020) (Figure 3).

## 4. Discussion

In this study, we investigated whether SAA is associated with the clinical features of AAV and found several interesting results. First, SAA was well correlated with BVAS, FFS, SF-36, and acute phase reactants. Second, high BVAS and poor HRQoL could be predicted by setting an optimal cut-off value of SAA. Third, SAA > 1173.6 ng/mL was found as an independent predictor of high BVAS in an adjusted logistic regression analysis, together with serum albumin. Fourth, when the patients were classified into two groups according to the optimal cut-off value of SAA, patients with SAA ≥ 1173.6 ng/mL exhibited a significantly higher risk of having high BVAS and poor HRQoL compared to those without (RR 3.419 and 1.493). Taken together with these results, we concluded that SAA could be a useful biomarker in estimating disease activity and HRQoL in AAV.

We hypothesize that SAA could be relevant to the cross-sectional disease activity or severity of AAV based on the fact that SAA could influence neutrophil activation and the production of key proinflammatory cytokines implicated in AAV. First, SAA might enhance the recruitment of neutrophils, which are important immune cells in the pathogenesis of AAV [20]. SAA could bind to formyl peptide receptor 2 (FPR2) on the cell membrane of neutrophils and play as a chemoattractant driving them to the inflamed tissues [21]. In addition, SAA could bind to FPR2 on the cell membrane of monocytes and augment the production of C-X-C motif chemokine ligand 8 (CXCL8). Subsequently, secreted CXCL8 from monocytes could bind to C-X-C motif chemokine receptor 2 on the cell membrane of neutrophils and also drive neutrophils to the inflamed tissues in a synergistic way [22]. Second, SAA is capable of forming a vicious cycle of proinflammatory cytokine and chemokine production. SAA stimulates various immune cells and tissue-specific cells and drives them to produce proinflammatory cytokines of IL-1*β*, IL-6, and TNF-*α* [23]. These cytokines, which are also thought to play an important role in AAV, can increase the production of SAA from the liver, resulting in continuing a vicious cycle between SAA and the cytokine/chemokine network [10, 24, 25].

In the present study, we observed that the SAA level was higher in AAV cases of lung and kidney involvement compared to those without. AAV is a vasculitis mainly affecting capillaries and adjacent arterioles and venules, and the lungs and kidneys are organs in which capillaries are predominantly present in order to exchange air and excrete metabolic products in the body efficiently. For this reason, the inflammatory burden of capillaritis in the lungs and kidneys might be expected to be much higher than other organs. Therefore, it may be speculated that compared to other organs, capillaritis in the lungs and kidneys provokes a higher amount of the proinflammatory cytokine, which in turn accelerates the production of SAA in the liver, leading to an increase in circulating concentration of SAA. However, because AAV patients with mucous membrane and eye involvement had the highest level of SAA, the organ-specific relationship between SAA in AAV should be further investigated.

Since SAA is primarily produced in the liver, SAA may be affected by liver diseases in theory [10]. A previous study reported that SAA-inducing cytokines were upregulated by hepatitis C virus (HCV) [26]. Thus, it could be speculated that increased circulating SAA may be influenced by both the inflammatory burden of AAV and hepatitis. However, in terms of the correlation between SAA and liver enzymes of aspartate aminotransferase (AST) and alanine aminotransferase (ALT), SAA was not significantly correlated with both AST and ALT. Since there was no significant correlation between SAA and liver enzymes, and we excluded patients with chronic hepatitis B virus and HCV infection, it can be suggested that the elevated SAA level is associated with the inflammatory burden of AAV in this study.

It is also intriguing that SAA is inversely correlated with both the physical and mental domains of SF-36, which is a conventional index to measure HRQoL. In the ROC analysis, SAA was revealed to be moderately accurate (AUROC 0.7-0.9) in discriminating poor and high HRQoL as well as in assessing disease activity [27]. In line with our observation, it was reported that SAA correlates with HRQoL measures in systemic sclerosis and could be a predictor of patient-reported outcome response in patients with rheumatoid arthritis [28, 29]. However, the HRQoL in patients with chronic diseases could be influenced by multiple factors such as disease severity and the use of medications such as glucocorticoids [30–32]. Nevertheless, the AUROC of SAA in predicting poor QoL (AUROC 0.729) was higher than that of BVAS (AUROC 0.690) and representative acute phase reactants ESR and CRP (AUROC 0.562 and 0.710). In addition, when we compared SF-36 PCS and MCS scores between current steroid users and nonusers to exclude the effect of medications, no difference was observed, implying that SAA could provide clinically relevant information in assessing HRQoL in patients with AAV.

This study has a strength; that is, we demonstrated the predictive potential of SAA for the cross-sectional disease activity and HRQoL in AAV for the first time. However, our study also has several limitations. First, the number of patients included in our study is quite small. Second, the mechanism by which SAA is associated with the disease activity of AAV has not been addressed. Third, the results of the serial SAA measurement were not provided. Therefore, it is necessary to identify the clinical significance of SAA in AAV through a larger and well-designed study.

## 5. Conclusion

In conclusion, we showed that SAA was positively correlated with BVAS and SF-36 scores and significantly increased in AAV patients with high disease activity and poor HRQoL. Our results indicate that SAA might be a useful biomarker in assessing disease activity and HRQoL in AAV.

## Figures and Tables

**Table 1:** Clinical and laboratory data of the 75 patients.

	Values
Variants (*N* (%))	
MPA	38 (50.7%)
GPA	21 (28.0%)
EGPA	16 (21.3%)
Demographic data	
Age (years)	58.9 ± 15.1
Male gender (*N* (%))	26 (34.7%)
Disease duration (months)	18.2 ± 32.2
AAV-specific indices	
BVAS	9.6 ± 6.8
FFS (2009)	1.3 ± 0.9
VDI	3.2 ± 1.7
SF-36 PCS score	49.9 ± 23.3
SF-36 MCS score	56.8 ± 21.5
Clinical manifestations (*N* (%))	
General	26 (34.7%)
Cutaneous	9 (12.0%)
Mucous membrane and eye	5 (6.7%)
Ear, nose, and throat	35 (46.7%)
Pulmonary	48 (64.0%)
Cardiovascular	5 (6.7%)
Abdominal	0 (0.0%)
Renal	38 (50.7%)
Nervous system	16 (21.3%)
ANCA positivity (*N* (%))	
P-ANCA	41 (54.7%)
C-ANCA	7 (9.3%)
MPO-ANCA	37 (49.3%)
PR3-ANCA	6 (8.0%)
ANCA positivity	49 (65.3%)
Laboratory data	
WBC count (/mm^3^)	8.9 ± 4.4
Hemoglobin (g/dL)	11.6 ± 2.4
Platelet count (×1000/mm^3^)	303.3 ± 141.6
Blood urea nitrogen (mg/dL)	24.8 ± 16.7
Creatinine (mg/dL)	1.7 ± 1.8
Total protein (g/dL)	6.3 ± 0.7
Serum albumin (g/dL)	3.6 ± 0.7
AST (IU/L)	20.9 ± 12.8
ALT (IU/L)	24.4 ± 20.9
ESR (mm/hr)	44.6 ± 33.3
CRP (mg/L)	19.4 ± 39.8
SAA (ng/mL)	876.0 ± 825.0

Values are expressed as mean ± standard deviation or number (percentage). MPA: microscopic polyangiitis; GPA: granulomatosis with polyangiitis; EGPA: eosinophilic granulomatosis with polyangiitis; AAV: ANCA-associated vasculitis; ANCA: antineutrophil cytoplasmic antibody; BVAS: Birmingham Vasculitis Activity Score; FFS: five-factor score; VDI: vasculitis damage index; SF-36: Short-Form 36-Item Health Survey; PCS: physical component summary; MCS: mental component summary; P: perinuclear; C: cytoplasmic; MPO: myeloperoxidase; PR3: proteinase 3; WBC: white blood cell; AST: aspartate aminotransferase; ALT: alanine aminotransferase; ESR: erythrocyte sedimentation rate; CRP: C-reactive protein; SAA: serum amyloid A.

**Table 2:** Correlation of SAA with AAV-specific indices and laboratory data.

	Correlation coefficient (*r*)	*p* value
BVAS	0.642	<0.001
FFS	0.367	0.001
VDI	0.136	0.243
SF-36 PCS score	-0.456	<0.001
SF-36 MCS score	-0.394	<0.001
WBC count	0.292	0.011
Hemoglobin	-0.569	<0.001
Platelet count	0.324	0.005
Blood urea nitrogen	0.350	0.002
Creatinine	0.317	0.006
Total protein	-0.292	0.011
Serum albumin	-0.700	<0.001
AST	0.206	0.077
ALT	0.222	0.055
ESR	0.611	<0.001
CRP	0.629	<0.001

SAA: serum amyloid A; AAV: ANCA-associated vasculitis; ANCA: antineutrophil cytoplasmic antibody; BVAS: Birmingham Vasculitis Activity Score; FFS: five-factor score; VDI: vasculitis damage index; SF-36: Short-Form 36-Item Health Survey; PCS: physical component summary; MCS: mental component summary; WBC: white blood cell; AST: aspartate aminotransferase; ALT: alanine aminotransferase; ESR: erythrocyte sedimentation rate; CRP: C-reactive protein.

**Table 3:** Comparison of clinical and laboratory features according to BVAS.

	Patients with low BVAS (*N* = 37)	Patients with high BVAS (*N* = 38)	*p* value
Variants (*N* (%))			0.264
MPA	16 (43.2%)	22 (57.9%)	
GPA	12 (32.4%)	9 (23.7%)	
EGPA	9 (24.3%)	7 (18.4%)	
Demographic data			
Age (years)	59.7 ± 13.2	58.1 ± 16.8	0.644
Male gender (*N* (%))	11 (29.7%)	15 (39.5%)	0.379
Disease duration (months)	29.7 ± 37.0	6.9 ± 21.8	0.002
AAV-specific indices			
BVAS	4.0 ± 1.9	15.1 ± 5.2	<0.001
FFS (2009)	1.2 ± 0.8	1.4 ± 1.0	0.221
VDI	3.0 ± 1.9	3.4 ± 1.6	0.263
SF-36 PCS score	57.4 ± 23.2	42.6 ± 21.3	0.005
SF-36 MCS score	61.8 ± 20.0	52.0 ± 22.1	0.048
Clinical manifestations (*N* (%))			
General	6 (16.2%)	20 (52.6%)	0.001
Cutaneous	3 (8.1%)	6 (15.8%)	0.480
Mucous membrane and eye	1 (2.7%)	4 (10.5%)	0.358
Ear, nose, and throat	16 (43.2%)	19 (50.0%)	0.560
Pulmonary	19 (51.4%)	29 (76.3%)	0.025
Cardiovascular	2 (5.4%)	3 (7.9%)	0.999
Abdominal	0 (0.0%)	0 (0.0%)	N/A
Renal	13 (35.1%)	25 (65.8%)	0.008
Nervous system	8 (21.6%)	8 (21.1%)	0.952
ANCA positivity (*N* (%))			
P-ANCA	16 (43.2%)	25 (65.8%)	0.051
C-ANCA	2 (5.4%)	5 (13.2%)	0.430
MPO-ANCA	13 (35.1%)	24 (63.2%)	0.016
PR3-ANCA	1 (2.7%)	5 (13.2%)	0.200
ANCA positivity	19 (51.4%)	30 (78.9%)	0.013
Laboratory data			
WBC count (/mm^3^)	7.5 ± 3.4	10.4 ± 4.9	0.004
Hemoglobin (g/dL)	12.6 ± 1.8	10.7 ± 2.6	<0.001
Platelet count (×1000/mm^3^)	256.8 ± 76.7	348.7 ± 173.5	0.004
Blood urea nitrogen (mg/dL)	19.5 ± 10.3	29.9 ± 20.0	0.006
Creatinine (mg/dL)	1.2 ± 1.1	2.3 ± 2.1	0.008
Total protein (g/dL)	6.6 ± 0.6	6.1 ± 0.7	0.004
Serum albumin (g/dL)	4.0 ± 0.4	3.2 ± 0.6	<0.001
AST (IU/L)	21.4 ± 14.0	19.9 ± 8.8	0.609
ALT (IU/L)	23.0 ± 20.2	25.7 ± 21.7	0.578
ESR (mm/hr)	31.4 ± 17.7	57.4 ± 39.6	<0.001
CRP (mg/L)	3.8 ± 6.5	34.5 ± 51.5	<0.001
SAA (ng/mL)	423.1 ± 481.8	1317.1 ± 854.7	<0.001

Values are expressed as mean ± standard deviation or number (percentage). BVAS: Birmingham Vasculitis Activity Score; MPA: microscopic polyangiitis; GPA: granulomatosis with polyangiitis; EGPA: eosinophilic granulomatosis with polyangiitis; AAV: ANCA-associated vasculitis; ANCA: antineutrophil cytoplasmic antibody; FFS: five-factor score; VDI: vasculitis damage index; SF-36: Short-Form 36-Item Health Survey; PCS: physical component summary; MCS: mental component summary; N/A: not applicable; P: perinuclear; C: cytoplasmic; MPO: myeloperoxidase; PR3: proteinase 3; WBC: white blood cell; AST: aspartate aminotransferase; ALT: alanine aminotransferase; ESR: erythrocyte sedimentation rate; CRP: C-reactive protein; SAA: serum amyloid A.

**Table 4:** Comparison of SAA between patients according to the presence of organ involvement.

Clinical manifestation	SAA (ng/mL)		*p* value
	Yes	No	
General	1425.8 ± 754.5	584.3 ± 708.7	<0.001
Cutaneous	860.1 ± 879.6	878.2 ± 824.4	0.951
Mucous membrane and eye	1948.7 ± 215.6	799.4 ± 798.7	0.002
Ear, nose, and throat	810.9 ± 810.9	933.0 ± 843.3	0.526
Pulmonary	1030.0 ± 859.1	602.3 ± 695.0	0.030
Cardiovascular	896.8 ± 669.7	874.5 ± 839.0	0.954
Renal	1155.0 ± 840.6	589.5 ± 711.7	0.002
Nervous system	562.6 ± 690.9	961.0 ± 842.9	0.087

Values are expressed as mean ± standard deviation. SAA: serum amyloid A.

**Table 5:** Univariate and multivariate logistic regression analyses for the prediction of high BVAS.

	Univariate analysis			Multivariate analysis		
	OR	95% CI	*p* value	OR	95% CI	*p* value
P-ANCA	2.524	0.992-6.422	0.052			
MPO-ANCA	3.165	1.232-8.130	0.017			
ANCA positivity	3.553	1.292-9.772	0.014			
WBC count	1.211	1.052-1.393	0.008			
Hemoglobin	0.690	0.552-0.862	0.001			
Blood urea nitrogen	1.047	1.010-1.085	0.012			
Creatinine	1.511	1.083-2.108	0.015			
Total protein	0.351	0.162-0.759	0.008			
Serum albumin	0.063	0.017-0.239	<0.001	0.132	0.032-0.551	0.006
ESR	1.030	1.011-1.049	0.002			
CRP	1.071	1.007-1.138	0.030			
SAA > 1173.6 ng/mL	33.654	6.969-162.527	<0.001	15.132	2.656-86.196	0.002

BVAS: Birmingham Vasculitis Activity Score; P: perinuclear; ANCA: antineutrophil cytoplasmic antibody; MPO: myeloperoxidase; WBC: white blood cell; BUN: blood urea nitrogen; ESR: erythrocyte sedimentation rate; CRP: C-reactive protein; SAA: serum amyloid A.

## Data Availability

The raw data supporting the conclusions of this article will be made available by the authors, without undue reservation.
